# Pyrrolidine Dithiocarbamate (PDTC) Attenuates Cancer Cachexia by Affecting Muscle Atrophy and Fat Lipolysis

**DOI:** 10.3389/fphar.2017.00915

**Published:** 2017-12-12

**Authors:** Chunxiao Miao, Yuanyuan Lv, Wanli Zhang, Xiaoping Chai, Lixing Feng, Yanfen Fang, Xuan Liu, Xiongwen Zhang

**Affiliations:** ^1^Shanghai Engineering Research Center of Molecular Therapeutics and New Drug Development, College of Chemistry and Molecular Engineering, East China Normal University, Shanghai, China; ^2^Institute of Interdisciplinary Integrative Biomedical Research, Shanghai University of Traditional Chinese Medicine, Shanghai, China; ^3^Division of Anti-tumor Pharmacology, Shanghai Institute of Materia Medica (CAS), Chinese Academy of Sciences, Shanghai, China

**Keywords:** cancer cachexia, PDTC, C2C12 myotubes, 3T3-L1 adipocytes, muscle atrophy, fat lipolysis

## Abstract

Cancer cachexia is a kind of whole body metabolic disorder syndrome accompanied with severe wasting of muscle and adipose tissue. NF-κB signaling plays an important role during skeletal muscle atrophy and fat lipolysis. As an inhibitor of NF-κB signaling, Pyrrolidine dithiocarbamate (PDTC) was reported to relieve cancer cachexia; however, its mechanism remains largely unknown. In our study, we showed that PDTC attenuated cancer cachexia symptom in C26 tumor bearing mice models *in vivo* without influencing tumor volume. What’s more, PDTC inhibited muscle atrophy and lipolysis in cells models *in vitro* induced by TNFα and C26 tumor medium. PDTC suppressed atrophy of myotubes differentiated from C2C12 by reducing MyoD and upregulating MuRF1, and preserving the expression of perilipin as well as blocking the activation of HSL in 3T3-L1 mature adipocytes. Meaningfully, we observed that PDTC also inhibited p38 MAPK signaling besides the NF-κB signaling in cancer cachexia *in vitro* models. In addition, PDTC also influenced the protein synthesis of skeletal muscle by activating AKT signaling and regulated fat energy metabolism by inhibiting AMPK signaling. Therefore, PDTC primarily influenced different pathways in different tissues. The study not only established a simple and reliable screening drugs model of cancer cachexia *in vitro* but also provided new theoretical basis for future treatment of cancer cachexia.

## Introduction

Cachexia is a severe wasting syndrome accompanied with serious loss of body weight during a lot of chronic diseases such as cancer, AIDS, tuberculosis (Tisdale, 2009). Cancer cachexia affects about 50–80% of cancer patients and is mainly characterized by fatigue, loss of muscle and fat mass, excessive consumption of energy and systemic inflammation (Fearon et al., 2011; von Haehling and Anker, 2014). Cancer cachexia not only influences patients’ quality of life, but also weakens the efficacy of chemotherapy and radiotherapy on tumor, therefore decreasing patients’ survival time seriously (Kumar et al., 2010). It is believed that cancer cachexia is responsible for death of more than 20% of cancer patients directly and indirectly (Fearon et al., 2013; von Haehling and Anker, 2014).

Given the detrimental clinical consequences, it is mandatory to relieve and/or delay the progression of cancer cachexia. At present, there is no approved therapeutic agent for the treatment or prevention of cancer cachexia. A variety of therapeutics including nutritional supplementation, appetite stimulation, and anti-inflammatory strategies has been used to manage cancer cachexia symptoms. Omega-3 Fatty Acids were investigated in clinical Phase I/II to test whether it could help body weight stabilization in cancer cachexia (Harle et al., 2005; Yeh et al., 2013). Anamorelin, a ghrelin receptor agonist, was applied to treat patients with non-small cell lung cancer (NSCLC) and cachexia–anorexia to enhance appetite and anabolic activity in clinical phase III (Garcia et al., 2015; Currow et al., 2017). MT-102 (Espindolol), a novel anabolic/catabolic transforming agent, was used to treat subjects with cachexia related to stage III and IV non-small cell lung cancer and colorectal cancer in clinical phase II. Infliximab, anti-TNFα monoclonal antibody, was applied to treat cancer-related cachexia in subjects with pancreatic cancer in clinical phase II (Wiedenmann et al., 2008; Arruda et al., 2010; Gueta et al., 2010; Miksza et al., 2013). Although these agents have entered into clinical evaluation, it is increasingly evident that a single therapy may not be sufficient to prevent or ameliorate cancer cachexia due to the complexity of this syndrome. Therefore, better understanding the molecular mechanisms of cancer cachexia will allow the identification of potential therapeutic targets and the development of promising drugs.

NFκB signaling plays an important role in skeletal muscle atrophy and fat lipolysis. NF-κB suppressed MyoD mRNA at the post-transcriptional level and upregulated the expression of MuRF1 in muscle decay and cachexia (Li and Reid, 2000; Bodine et al., 2001; Vallabhapurapu and Karin, 2009). And, TNF-α-mediated lipolysis was reduced in the presence of NF-κB inhibitor (Laurencikiene et al., 2007). Therefore, NF-κB inhibitors, such as Compound A, DHMEQ, curcumin, resveratrol, and SN50, were used to keep the mass of skeletal muscle and fat and even inhibit tumor growth. Compound A only partially rescues the phenotype of the cachectic gastrocnemius on the level of metabolism (Der-Torossian et al., 2013). DHMEQ could prevent the development of cachexia in JCA-1 tumor-bearing mice presumably through the inhibition of IL-6 secretion (Kuroda et al., 2005). SN50 inhibited the expression of proteasome induced by PIF (proteolysis-inducing factor) to relieve muscle wasting in cancer cachexia (Wyke et al., 2004). Curcumin completely attenuated total protein degradation in murine myotubes induced by PIF. However, it was ineffective in preventing loss of body weight of MAC16 tumor bearing mice (Wyke et al., 2004). Resveratrol was accompanied by inhibition of tumor growth while attenuating weight loss (Wyke et al., 2004).

**FIGURE 1:**
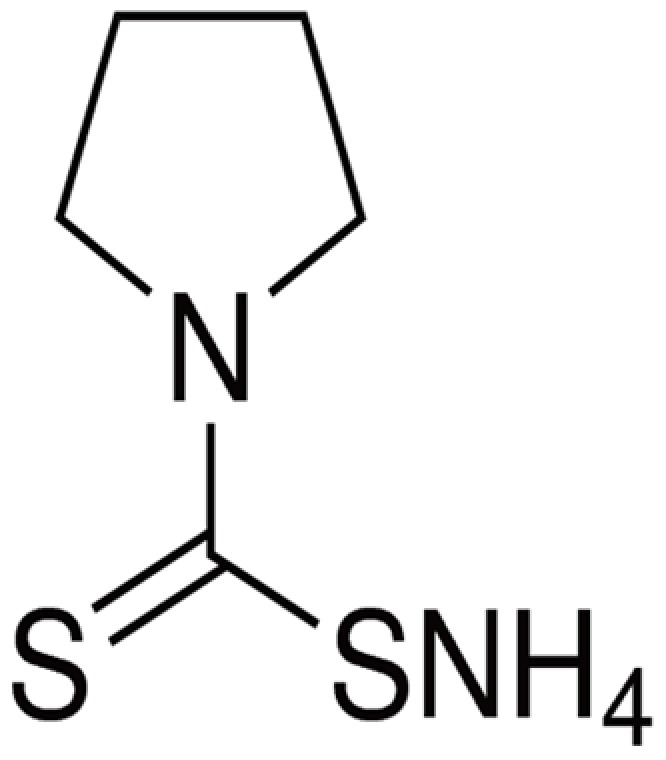
Chemical structure of Pyrrolidine dithiocarbamate (PDTC).

Pyrrolidine dithiocarbamate (PDTC, **Figure 1**), a STAT/NF-κB inhibitor and an antioxidant, is known to exert anti-inflammation, antioxidant, and radical scavenger functions (Tahata et al., 2014). Recently, the effect of PDTC on attenuating cachexia has attracted much attention. Nai et al. (2007) reported that PDTC could attenuate the development of cancer cachexia in C26 tumor-bearing mice by inhibiting the increase of IL-6 levels in serum and tumor tissue as well as inhibiting NF-κB activation in the tumor sites. In consistence, administration of PDTC also relieved cancer cachexia in Lewis lung carcinoma (LLC) tumor-bearing mice. PDTC reduced muscles STAT3 and p65 phosphorylation, but did not alter LLC-induced muscles AMPK or AKT phosphorylation (Puppa et al., 2014a). Moreover, in the study of Narsale *et al.*, PDTC neither suppressed the cachexia induction of plasma IL-6, nor affected the cachexia-enhanced phosphorylation of NF-κB (S468) in skeletal muscle. The inhibitory effect of PDTC on cancer cachexia was further confirmed in *APC^Min/+^* mouse, which exhibited an IL-6-dependent cachexia and had long duration of cachexia development. PDTC suppressed the cachexia induction of STAT3 activation and increased mTORC1 signaling in muscle, while attenuated glycogen and lipid content depletion independent to the activation of STAT3 and mTORC1 signaling in liver. (Narsale et al., 2016). Collectively, these studies demonstrated that PDTC exhibited potential activity against cancer cachexia, but its mechanisms could not be simply attributed to the inhibitory effect of PDTC on NF-κB signaling. Moreover, signaling responses to PDTC in different tissues might be different, which also deserved further evaluation.

In the present study, we systematically determined effects of PDTC on cancer cachexia in C26 tumor bearing mice *in vivo* and *in vitro*, and studied the signaling pathways involved in protein turnover in skeletal muscle atrophy and lipolysis in adipocytes to thoroughly elucidate the mechanisms of PDTC on relieving cancer cachexia.

## Materials and Methods

### Reagents

PDTC purchased from Sigma–Aldrich (St. Louis, MO, United States) was reconstituted in sterile saline and stored at -20°C. RIPA Lysis and Halt Protease and Phosphatase Inhibitor Cocktail (100×) were purchased from Thermo Scientific (Rockford, IL, United States) and stored at 4°C. BCA protein assay kit used to quantify protein concentration were purchased from Beyotime (Shanghai, China) and stored at RT. DMEM (High Glucose), Penicillin/streptomycin and Trypsin/EDTA were purchased from Hyclone (Los Angeles, CA, United States). Horse serum was purchased from Gbico (New York, NY, United States). Fetal bovine serum (FBS) was derived from Biological Industries (Kibbutz Beit Haemek, Israel). TNFα was purchased from PeproTech (Rocky Hill, CT, United States).

### Animals

All animal (purchased from Shanghai SLAC Laboratory Animal, Co., Ltd., Shanghai, China) care and experimental protocols for this study complied with the Chinese regulations and the Guidelines for the Care and Use of Laboratory Animals drawn up by the National Institutes of Health (United States) and were approved by the Institutional Animal Care and Use Committee of the East China Normal University. Male BALB/c mice (6–8 weeks old) were purchased from the Shanghai SLAC Laboratory Animal CO. LTD. Mice were maintained on a 12:12 light–dark cycle in a temperature-controlled (21∼23°C) and specific pathogen-free (SPF) conditional room, and were provided standard rodent chow and water *ad libitum*. All animals were acclimatized for a week before beginning the study.

### Cancer Cachexia Model *in Vivo*

Male BALB/c mice with same initial body weight were randomly divided into four groups (12 mice per group): health group (without tumor), Colon-26 (C26, obtained from Shanghai Institute of Materia Medica, Chinese Academy of sciences) tumor-bearing mice group (C26 model group) and C26 tumor-bearing mice treated with PDTC (50, 100 mg/kg) group (Nai et al., 2007). On day 0, mice were implanted subcutaneously in the right flank with 100 μl (1.0 × 10^6^) C26 adenocarcinoma cells. Starting from the next day, C26 model group mice received daily intraperitoneal injections of sterile saline, while PDTC treated mice received daily intraperitoneal injections of PDTC (50, 100 mg/kg). Body weight, tumor volume, and food intake were measured daily from inoculation to completion of the study. On day 6, tumors were first noticed. Record the shortest diameter (*x*) and longest diameter (*y*) of tumor using calipers. Tumor volume was calculated following the formula: *V* = *x* ^∗^ *x* ^∗^ *y* ^∗^ 0.5. When the mice lost 10% of their body weight or when their tumor volumes reached 2,000 mm^3^, tumor, gastrocnemius muscles and eWAT (epididymal white fat) tissue were rapidly dissected, weighed, and frozen in liquid nitrogen, then stored at –80°C until ready for further analyses, or fixed in 4% paraformaldehyde overnight and embedded in paraffin. All treatment groups were sacrificed by cervical dislocation under ether anaesthesia 6 h after the last treatment.

### Cell Culture

C26 adenocarcinoma cells were maintained in RMPI-1640 medium (Hyclone, Los Angeles, CA, United States) containing 10% FBS at 37°C with 5% CO_2_.

C2C12 murine myoblast cell line, obtained from ATCC, were cultured in high-glucose DMEM with 10% FBS at 37°C with 5% CO_2_. During differentiation, the medium of cells planted on culture plates coated with 0.1% gelatin was switched into differentiation medium (high-glucose DMEM containing 2% horse serum) when cell confluence reached 70%. After 5 days, multinuclear myotubes were formed.

3T3-L1 pre-adipocytes cells, obtained from Shanghai Institute of Materia Medica, Chinese Academy of Sciences, were cultured in adipocytes medium (AM, high-glucose DMEM with 10% FBS) at 37°C with 5% CO_2_. During differentiation, the pre-adipocytes were planted on culture plates coated with 0.1% gelatin, with confluence reached 100% for 48 h in AM. Then they were induced to differentiate by treatment with differentiation media (DM I and DM II) for 48 h, respectively, DM I containing 10 μg/ml insulin (Solarbio, Beijing, China), 1 μM dexamethasone (DEX, Sigma–Aldrich, St. Louis, MO, United States) and 0.5 mM 3-isobutyl-1-methylxanthine (IBMX, Sigma–Aldrich, St. Louis, MO, United States) in AM and DM II (DEX- and IBMX-free DM I). Thereafter, the differentiated cells were maintained in AM changed in every 2 days until used (Chaiittianan et al., 2017).

All cells were negative for mycoplasma contamination before use.

### C26 Tumor Medium Collection

When C26 tumor cells confluence reached 70%, the medium was switched into new high-glucose DMEM medium for 48 h. Thereafter, medium was collected and centrifuged at 5000 *g* for 10 min at 4°C. Medium from non-tumor cells (C2C12 cell or 3T3-L1 cell) was used as control medium. The final supernatant was filtered and stored at –20°C or used immediately at a 1:1 dilution with fresh normal medium.

### Cancer Cachexia Models *in Vitro*

C2C12 myotubes cells were incubated with TNFα (100 ng/ml) or 50% C26 tumor medium in 2% horse serum in high-glucose DMEM for 48 h in the presence of PDTC or sterile saline as control reagents. Then cells were harvested for Western Blotting or used for morphological analysis.

3T3-L1 mature adipocytes were incubated with TNFα (50 ng/ml) or 50% C26 tumor medium in 10% FBS in high-glucose DMEM for 48 h in the presence of PDTC or sterile saline as control reagents. Then cells were harvested for Western Blotting or used for morphological analysis.

### Western Blot

Western blots were performed as described previously (Hetzler et al., 2015). Briefly, C2C12 myotubes and 3T3-L1 mature adipocytes were homogenized in RIPA buffer plus a phosphatase protease inhibitor. The lysates were centrifuged at 13000 rpm for 30 min at 4°C. The supernatant was quantified for protein concentration using the BCA Protein Assay Kit (Beyotime, Shanghai, China). Equal amounts of protein samples were separated by 10% SDS-PAGE gel electrophoresis and transferred to a polyvinylidene fluoride membrane. The PVDF membranes were blocked in 5% non-fat milk in phosphate buffered saline (PBST, containing 0.1% Tween 20) for 1 h at room temperature and then incubated with primary antibodies diluted in 5% BSA-TPBS at 4°C overnight. The primary antibodies used were as follows:MuRF-1 (1:1000, Proteintech), P38, MyoD (1:1000, Cell Signaling Technology, Beverly, MA, United States), P65 (1:1000, Cell Signaling Technology), p-P65 (1:1000, Cell Signaling Technology), AKT (1:500, Santa, Orange, CA, United States), p-AKT (1:2000, Cell Signaling Technology), AMPK (1:1000, Cell Signaling Technology), p-AMPK (1:1000, Cell Signaling Technology), P38 MAPK (1:1000, Cell Signaling Technology), p-P38 MAPK (1:1000, Cell Signaling Technology), Peri A antibodies (1:1000, Cell Signaling Technology), MHC (1:1000, DSHB, Iowa City, IA, United States) and GAPDH-HRP (1:5000, Santa Cruz Biotechnology, Dallas, TX, United States). Anti-mouse (1:5000, Multi Sciences, Hangzhou, China) and anti-rabbit (1:5000, Multi Sciences, Hangzhou, China) IgG horseradish peroxidase-conjugated secondary antibody was incubated with membranes for 1 h in 5% non-fat milk in TPBS. ECL Chemiluminescent Kit (Thermo Fisher, Waltham, MA, United States) was used to visualize the antibody-antigen interaction and chemical luminescence of membranes was detected by Amersham Imager 600 (GE).

### Hematoxylin-Eosin (HE) Staining

Gastrocnemius muscle samples and epididymal white adipose tissue (eWAT) were freshly isolated and fixed in 4% paraformaldehyde (PFA) for 24 h. Paraffin-embedded tissues were cut in 10 μm sections stained with hematoxylin and eosin (H&E) by standard procedures.

### Immunofluorescent Staining

Differentiated C2C12 myotubes were fixed by 4% PFA for 30 min at room temperature, permeabilized with 0.5% Triton X-100 in PBS for 10 min, and then blocked with 5% bovine serum albumin (BSA) in PBS for 1 h at room temperature. Myotubes were incubated with anti-MHC (MF-20, 1:100, DSHB) diluted in 5% BSA overnight at 4°C. Myotubes were incubated with secondary antibody Cy3-AffiniPure rabbit anti-mouse IgG (H+L) (1:500, Jackson) at room temperature. Images were captured by fluorescence microscope (Leica) and the diameter of myotubes was measured by Image J.

### Oil Red O Staining

Cells were washed three times with phosphate-buffered saline (PBS), fixed in 4% formalin for 30 min, and then washed three times with cold PBS. Cells were stained in the Oil Red O (Sigma–Aldrich, St. Louis, MO, United States) working solution (3:2, 0.5% Oil Red O dye in isopropanol: water) for 30 min at room temperature (25°C) and washed three times with water. Staining was visualized by bright-field microscopy.

### Triglyceride Isolation and Determination

Triglycerides (TG) was assessed through commercial enzymatic kits. Differentiated 3T3-L1 adipocytes were harvested in 100 μl distilled water containing 5% Triton-X100; and the TG levels were determined using a commercial kit (Triglyceride Quantification Kit, Applygen, Beijing, China) following the manufacturer instructions. TG of serum was assessed with Automatic biochemical analyzer (HITACHI 7020).

### Lipolysis Assays *in Vitro*

For lipolysis experiments, glycerol accumulation in the media from 3T3-L1 mature adipocytes and serum of mice was measured using a Lipolysis Assay Kit (Applygen) following the manufacturer instructions. Briefly, 3T3-L1 mature adipocytes were washed three times with PBS and incubated with 100 μl phenol red-free DMEM supplemented with 1% fatty acid-free BSA containing 50 ng/ml TNFα or 50% C26 tumor medium with or without PDTC for 24 h. After incubation, the 100 μl medium was collected and centrifuged at 12000 *g* for 10 min to remove cell debris. The 50 μl supernatant or serum of mice and glycerol assay reagent (150 μl) were plated in a clean 96-well plate for 10 min at 37°C and optical density of each well was measured at 550 nm.

### Statistical Analysis

Data are expressed as mean ± SEM. Two-tailed Student’s *t*-test was used for comparisons between two groups. One-way ANOVA test was performed to compare multiple groups followed by Bonferroni’s *post hoc* test. A *p*-value of 0.05 or lower was considered significant in all experiments. All analyses were performed using GraphPad Prism 5.0. Values of *p* less than 0.05 were considered to be statistically significant and were presented as ^∗^*p* < 0.05, ^∗∗^*p* < 0.01, ^∗∗∗^*p* < 0.001 or ^#^*p* < 0.05, ^##^*p* < 0.01, ^###^*p* < 0.001.

## Results

### PDTC Attenuates C26 Tumor-Induced Body Weight Loss *in Vivo*

**FIGURE 2:**
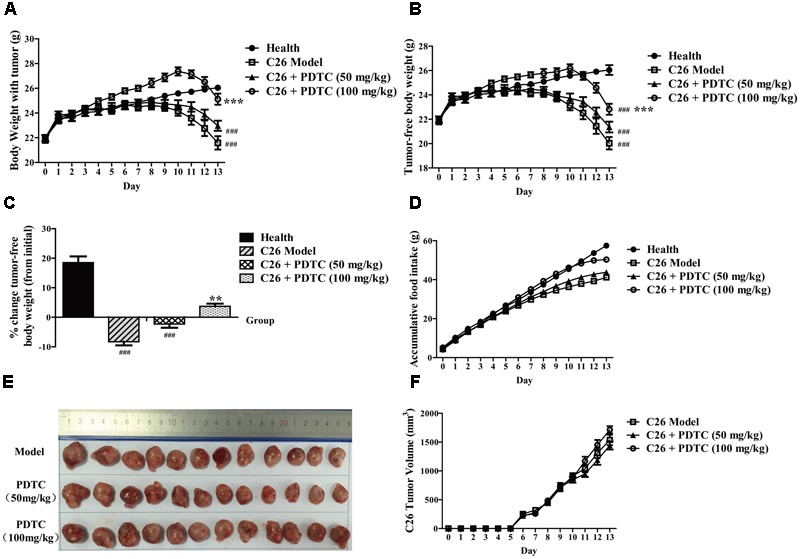
Pyrrolidine dithiocarbamate attenuates cachexia symptoms of C26 tumor-bearing mice. PDTC (50 and 100 mg/kg) was injected intraperitoneally daily (*n* = 12). **(A)** Body weight of mice. **(B)** Tumor-free body weight of mice. **(C)** Changes of tumor-free body weight of mice **(D)** Accumulative food intake of mice. **(E,F)** Tumor volume of mice. Data presented are the mean ± SE of three independent experiments. #Versus health group mice; ^∗^versus C26 tumor bearing group mice. One-way ANOVA test was performed followed by Bonferroni’s *post hoc* test. ^###^*p* < 0.001; ^∗∗^*p* < 0.01, ^∗∗∗^*p* < 0.001.

The effect of PDTC to attenuate cachexia in C26 tumor bearing mice was systematically evaluated in our experiment. In line with previous study, PDTC effectively suppressed C26 tumor-induced body weight loss. Mice in C26 model group and PDTC (50 mg/kg)-treated group started to lose body weight on day 9. In contrast, the weight loss of mice with PDTC (100 mg/kg) treatment was delayed for two days (on day 11). At the end of the treatment (day 13), the body weight of mice treated with PDTC (100 mg/kg) was significantly higher than that of C26 model group. The body weight of mice treated with PDTC (50 mg/kg) was also higher than that of C26 model group, even though there was no statistical significance (**Figure 2A** and **Table 1**). To avoid the influence of tumor weight on body weight, we also analyzed the tumor-free body weight. The overall trend of tumor-free body weight recaptured the effect of PDTC presented by body weight (**Figure 2B** and **Table 1**). The changes of tumor-free body weight increased by 18.52% in healthy mice, decreased by 8.29 and 2.29% in C26 model group and in PDTC treatment group (50 mg/kg), respectively, but increased by 3.73% in PDTC treatment group (100 mg/kg) (**Figure 2C**). What’s more, PDTC also increased food intake of mice. Specifically, the food intake in PDTC (100 mg/kg) group was a little higher than that in C26 model group (**Figure 2D**). As a result, the body weight of mice in PDTC (100 mg/kg) group started to increase on day 4 (**Figure 2A**). In addition, PDTC didn’t influence C26 tumor growth in mice (**Figures 2E,F**). Together, these results demonstrated that PDTC effectively attenuated C26 tumor-induced body weight loss, and did not affect C26 tumor growth.

**Table 1:** Effect of pyrrolidine dithiocarbamate (PDTC) treatment on parameters of healthy and C26-tumor bearing mice.

Group	*N*	Initial body weight(g)	Final body weight(g)	Final body weight without tumor(g)	Gastrocnemius muscle(g)	eWAT(g)
Health	13	22.0 ± 0.3	26.1 ± 0.2	26.1 ± 0.2	0.139 ± 0.003	0.477 ± 0.033
C26 Model	12	21.8 ± 0.3	21.6 ± 0.3^###^	20.0 ± 0.5^###^	0.107 ± 0.002^###^	0.094 ± 0.022^###^
C26+PDTC (50 mg/kg)	12	21.9 ± 0.2	23.0 ± 0.4^###^	21.4 ± 0.4^###^	0.112 ± 0.002^###^	0.156 ± 0.031^###^
C26+PDTC (100 mg/kg)	12	22.0 ± 0.2	25.1 ± 0.5^∗∗∗^	22.8 ± 0.5^###∗∗∗^	0.122 ± 0.003^###∗∗∗^	0.267 ± 0.033^###∗∗∗^

*Data presented are the mean ± SE. ^#^Versus health group mice; ^∗^versus C26 tumor bearing group mice. ^###^*p* < 0.001, ^∗∗∗^*p* < 0.001.*

### PDTC Reduces Loss of Skeletal Muscle and Adipose Tissue Mass *in Vivo*

**FIGURE 3:**
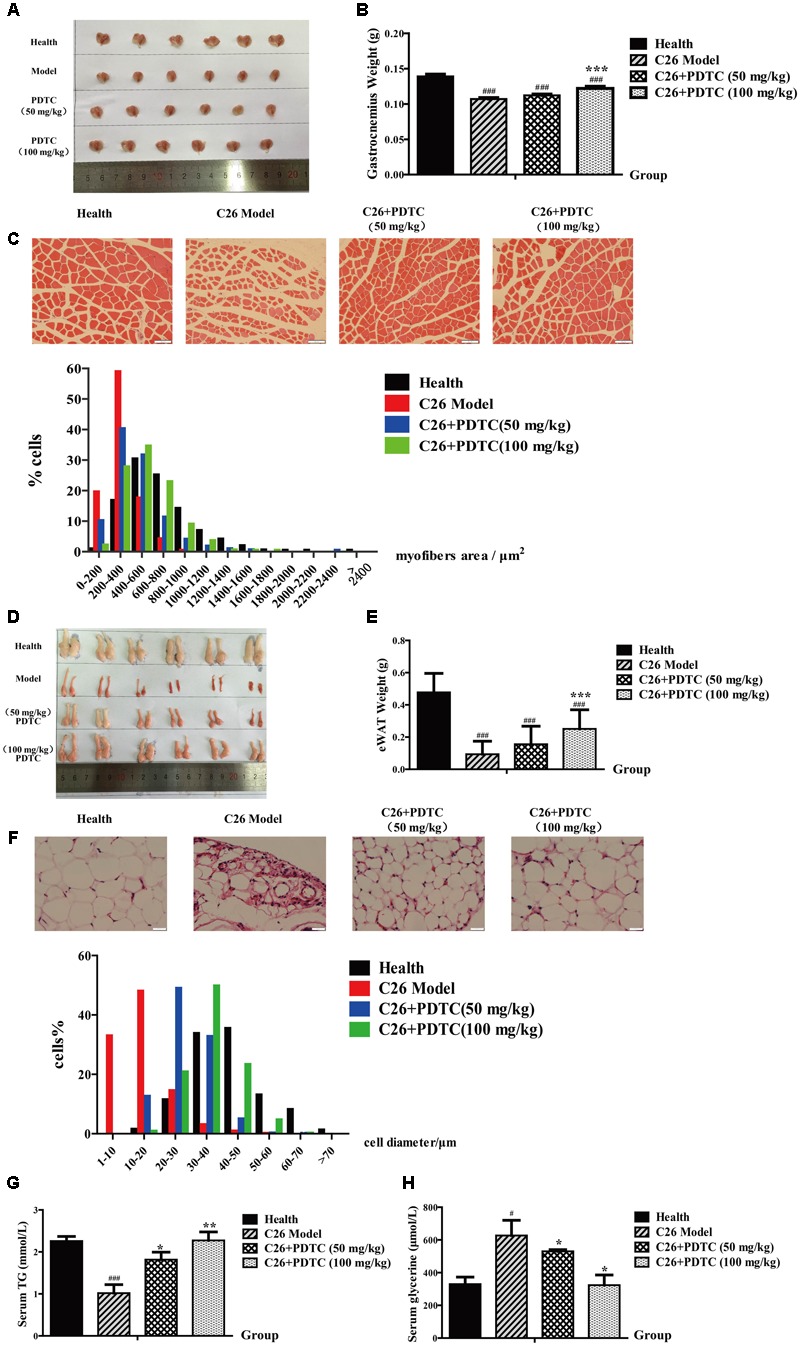
Pyrrolidine dithiocarbamate reduced the loss of skeletal muscle and fat in C26 tumor-bearing mice. PDTC (50 and 100 mg/kg) was injected intraperitoneally daily (*n* = 12). **(A,B)** GA weight of each group mice. **(C)** H&E-stained sections of mice GA and quantify the myofiber area of GA cell. **(D,E)** eWAT weight of each group mice. **(F)** H&E-stained sections of mice eWAT and quantify the diameter of adipocyte cell. **(G,H)** Content of TG and glycerol in serum. Scale bar of C, 50 μm. Scale bar of F, 20 μm. Data presented are the mean ± SE of three independent experiments. GA, gastrocnemius. eWAT, epididymal white fat. #Versus health group mice; ^∗^versus C26 tumor bearing group mice. One-way ANOVA test was performed followed by Bonferroni’s *post hoc* test. ^#^*p* < 0.05, ^##^*p* < 0.01, ^###^*p* < 0.001, ^∗^*p* < 0.05, ^∗∗^*p* < 0.01, ^∗∗∗^*p* < 0.001.

As cancer cachexia-induced weight loss is primarily from loss of skeletal muscle and body fat, we then analyzed the effect of PDTC on C26 tumor-induced loss of skeletal muscle and adipose tissue. As expected, C26 tumor led to a significant decrease of gastrocnemius (GA) mass, which was relieved by the treatment of PDTC (**Figures 3A,B** and **Table 1**). Comparing to C26 model mice, the change of GA mass increased by 4.7% in PDTC treatment group (50 mg/kg) and by 14.0% in PDTC treatment group (100 mg/kg), respectively (**Figure 3B** and **Table 1**). What’s more, PDTC also affected the myofibers size distribution. In healthy mice, a bell-like distribution of myofibers area was observed between 200 and1000 μm^2^. In contrast, the myofibers area of C26 model mice showed a smaller size distribution, with 80% cells distributed in less than 400 μm^2^. PDTC (50 and 100 mg/kg) treatment effectively reversed this shift and led the myofibers area redistributed between 200 and1000 μm^2^ (**Figure 3C**).

Similarly, PDTC also effectively inhibited the loss of body fat. Compared with healthy mice, C26 tumor caused a significant decrease of eWAT, which was relieved by the treatment of PDTC (**Figures 3D,E** and **Table 1**). Of note, the eWAT weights of PDTC treatment mice (100 mg/kg) were about 1.7-fold to that of C26 model mice (**Figure 3E** and **Table 1**). Moreover, PDTC also affected the size of adipocyte cell diameter. A bell-like distribution of adipocyte cell diameter was observed between 20 and 70 μm^2^ in healthy mice. However, a left shift was observed in C26 model with more than 80% adipocyte cells distributed in less than 20 μm^2^. PDTC obviously reversed this shift, which was in a dose-dependent manner (**Figure 3F**). In addition, the glycerol and TG content in mice serum further confirmed the protection of PDTC on lipolysis (**Figures 3G,H**). All these results supported that PDTC treatment attenuated the loss of body weight in C26 tumor-bearing cachexia mice by inhibiting GA atrophy and eWAT lipolysis in a dose-dependent manner (**Table 1**).

### PDTC Alleviates Muscle Atrophy in Cancer Cachexia Model *in Vitro*

**FIGURE 4:**
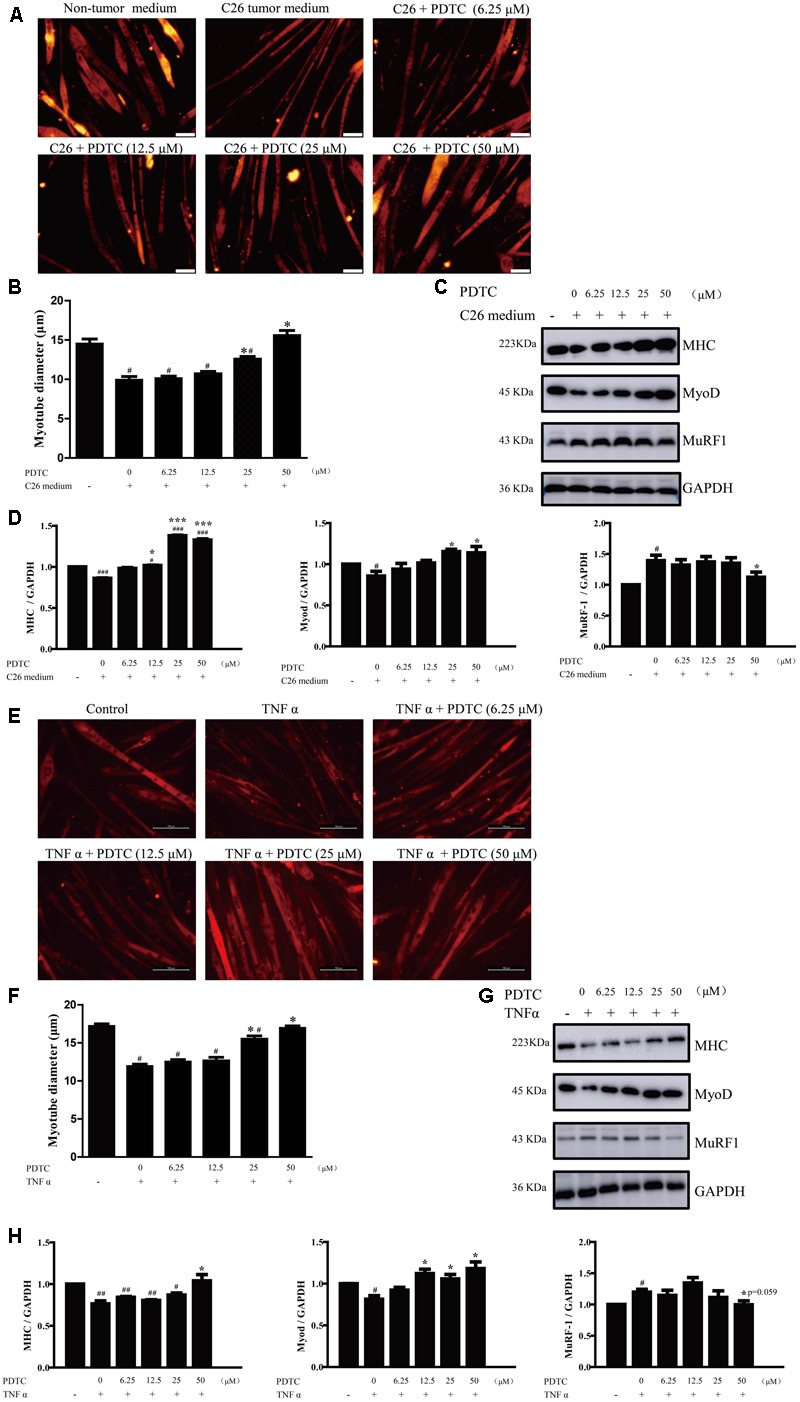
The effect of PDTC on C2C12 myotubes atrophy *in vitro*. The myotubes atrophy in cancer cachexia model *in vitro* was induced by C26-tumor medium (1:1 dilution with fresh normal medium) or TNFα (100 ng/ml) for 48 h. **(A)** The myotubes atrophy in cancer cachexia model induced by C26-tumor medium. **(B)** Quantified diameter of myotubes. **(C)** Representative Western blot of MHC, MyoD and MuRF-1 in cachexia model induced with C26-tumor medium. **(D)** The quantification of **(C)**. **(E)** The myotubes atrophy in cancer cachexia model induced by TNFα. **(F)** Quantified diameter of myotubes. **(G)** Representative Western blot of MHC, MyoD and MuRF-1 in cachexia model induced with TNFα. **(H)** The quantification of **(G)**. Scale bar of A, 50 μm. Scale bar of E, 100 μm. Data presented are the mean ± SE of three independent experiments. #Versus non-tumor medium (3T3-L1 cell medium) or control group; ^∗^versus C26-tumor medium or TNFα single treatment group. ^∗^*p* < 0.05, ^∗∗∗^*p* < 0.001, ^#^*p* < 0.05, ^##^*p* < 0.01, ^###^*p* < 0.001.

Multiple factors, including inflammation cytokines, decreased food intake and neuroendocrine changes (Oliff et al., 1987; Scott et al., 1996; Rivadeneira et al., 1999; Zhou et al., 2010; Sishi and Engelbrecht, 2011; Winbanks et al., 2016), contribute to the occurrence of cancer cachexia *in vivo*, which makes the mechanisms of cancer cachexia remain largely unknown. To make the question simple, here we used the *in vitro* system to investigate the mechanisms of PDTC on attenuating cancer cachexia. We first used C26 medium to induce atrophy of C2C12 myotubes and observed the protective effect of PDTC on myotubes atrophy. As shown in **Figure 4A**, C26 medium caused an obvious decrease of C2C12 myotubes diameter, and PDTC at high concentration (25 and 50 μM) effectively inhibited this decrease. In detail, the myotubes diameter decreased from 14.55 ± 0.58 μm in control cells to 9.95 ± 0.41 μm in C26 medium treated cells, but increased to 12.60 ± 0.31 μm and 15.62 ± 0.59 μm in the presence of 25 and 50 μM PDTC, respectively. Myosin Heavy Chain (MHC), a myogenic differentiation marker protein, is a preferred target of multiple pro-cachectic factors inducing muscle atrophy (Acharyya et al., 2004). Here we observed that C26 medium decreased the expression of MHC in C2C12 myotubes, whereas PDTC effectively reversed the downregulation of MHC at concentration of 25 and 50 μM. Moreover, the muscle differentiation factor MyoD and the ubiquitin ligase MuRF1 which affected the transcription and degradation of MHC, respectively, were determined in our experiment. Interestingly, C26 medium-induced downregulation of MyoD and upregulation of MuRF1 were suppressed by PDTC at concentration of 50 μM. The effect of PDTC on reversing myotubes atrophy was in a concentration-dependent manner (**Figures 4B–D**).

TNFα is one of the important factors involved in the pathogenesis of cancer cachexia; we then used TNFα to induce myotubes atrophy *in vitro*. Similarly, PDTC also efficiently inhibited TNFα-induced myotubes atrophy, which was in a concentration-dependent manner (**Figure 4E**). The myotubes diameter of cells treated with 50 μM PDTC was about 1.5-fold to that of TNFα treated cells (**Figure 4F**). PDTC also inhibited TNFα-induced downregulation of MHC and MyoD as well as up-regulation of MuRF1 (**Figures 4G,H**). The concentrations of PDTC used in these experiments had no cytotoxicity on viability of C2C12 myotubes (data not shown). These results demonstrated that PDTC protected the myotubes atrophy induced by C26 medium and TNF-α *in vitro*.

### Effect of PDTC on Inflammatory Signaling and Protein Synthesis of C2C12 Myotubes *in Vitro*

It is known that NF-κB upregulated the expression of MuRF1 (Li and Reid, 2000; Bodine et al., 2001; Vallabhapurapu and Karin, 2009) and suppressed MyoD mRNA at the posttranscriptional level in muscle decay and cachexia (Guttridge et al., 2000). Moreover, PDTC is an inhibitor of NF-κB and has different effect against the activity of NF-κB in different tissues. Therefore, we determined whether PDTC would inhibit the activation of NF-κB in C2C12 myotubes atrophy. The results showed that PDTC slightly inhibited C26 medium- induced phosphorylation of p65 (**Figures 6A,B**), which suggested that other signaling pathway was involved in the protective effect of PDTC against skeletal atrophy. Interestingly, we further found that PDTC significantly inhibited the enhanced phosphorylation of p38 MAPK in C26 medium-treated C2C12 myotubes. In addition, PDTC also increased the phosphorylation of AKT that was down-regulated after C26 medium treatment (**Figures 6C,D**). These results suggested that PDTC preserved the muscle mass by influencing the pathways of synthesis pathway and protein degradation.

### PDTC Attenuates Lipolysis in Cancer Cachexia Model *in Vitro*

**FIGURE 5:**
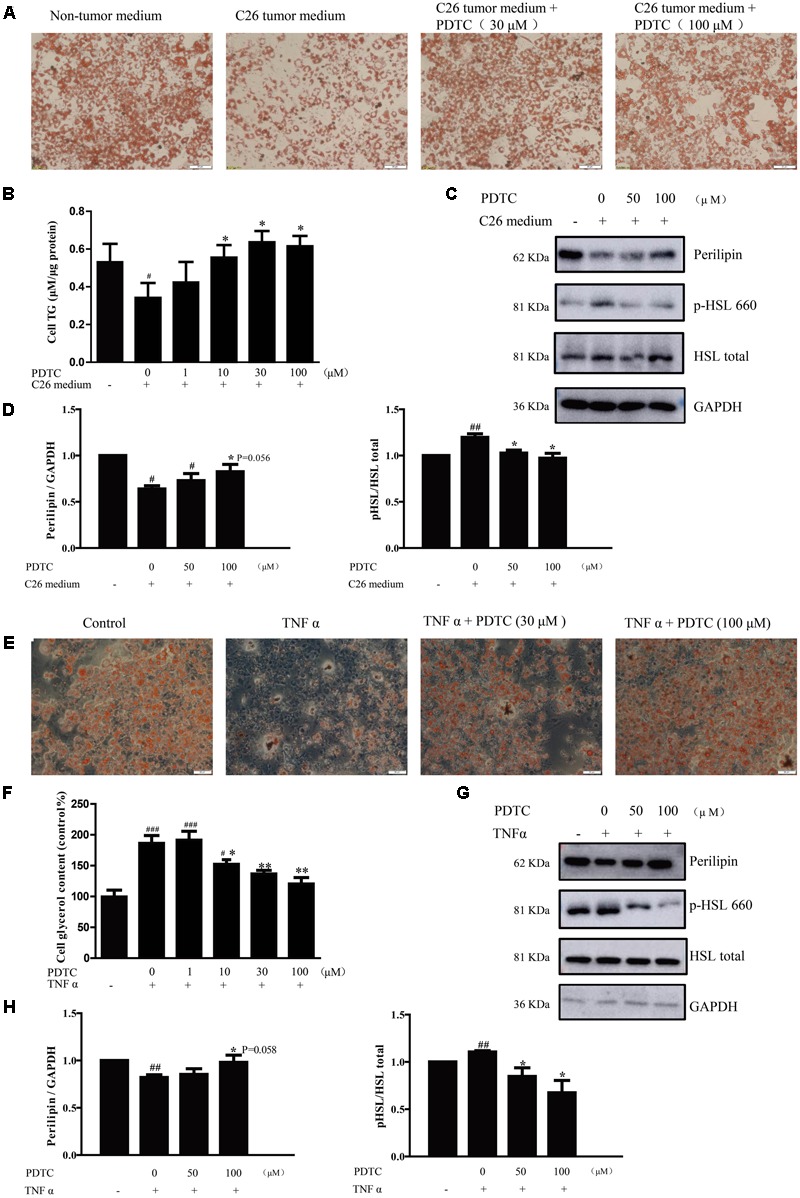
The effect of PDTC on lipolysis of 3T3-L1 adipocyte *in vitro*. The lipolysis of 3T3-L1 adipocyte in cancer cachexia model *in vitro* was induced by C26-tumor medium (1:1 dilution with fresh normal medium) or TNFα (50 ng/ml) for 48 h. **(A)** The lipid of 3T3-L1 mature adipocytes was detected by oil red O staining in cancer cachexia model induced by C26-tumor medium. **(B)** Quantified triglyceride (TG) with TG commercial kits. **(C)** Representative Western blot of phosphorylated HSL, total HSL and perilipin in cachexia model induced with C26-tumor medium. **(D)** The quantification of C. **(E)** The lipid of 3T3-L1 mature adipocytes was detected by oil red O staining in cancer cachexia model induced by TNFα. **(F)** Quantified glycerol release of medium with glycerol commercial kits. **(G)** Representative Western blot of phosphorylated HSL, total HSL and perilipin in cachexia model induced with TNFα. **(H)** The quantification of G. Scale bar, 20 μm. Data presented are the mean ± SE of three independent experiments. #Versus non-tumor medium (C2C12 cell medium) or control group; ^∗^versus C26-tumor medium or TNFα treatment group. ^∗^*p* < 0.05, ^∗∗^*p* < 0.01, ^#^*p* < 0.05, ^##^*p* < 0.01, ^###^*p* < 0.001.

In order to identify the effect of PDTC on lipolysis in cancer cachexia *in vitro*, we used C26 medium and TNFα to induce lipolysis of mature 3T3-L1 adipocytes. As shown by Oil Red O staining, the lipid of mature 3T3-L1 adipocytes with C26 medium treatment was much less than that with non-tumor medium (C2C12 cell medium). Meaningfully, C26 medium-induced decrease of lipid was suppressed in the presence of PDTC (**Figure 5A**). Consistent with this finding, PDTC effectively inhibited the decrease of TG content in 3T3-L1 adipocytes induced by C26 medium. In detail, the adipocyte TG content relative to cell lysis protein decreased from 0.53 ± 0.05 μM/μg protein in cells treated with non-tumor medium (C2C12 cell medium) to 0.34 ± 0.04 μM/μg protein in cells treated with C26 medium, and was reversed back to 0.42 ± 0.05, 0.56 ± 0.03, 0.64 ± 0.03, and 0.62 ± 0.03 μM/μg protein by the treatment of PDTC (1, 10, 30, and 100 μM, respectively). The effect of PDTC to inhibit C26 medium-induced decrease of TG content was in a dose-dependent manner (**Figure 5B**). We then determined the expression of perilipin that is a critical regulator of lipid stores in adipocytes (McDonough et al., 2013). In line with above findings, the expression of perilipin was remarkably down-regulated in mature 3T3-L1 adipocytes with C26 medium treatment, and this down-regulation was effectively suppressed by PDTC. Hormone sensitive lipase (HSL) is a rate-limiting enzyme that regulates adipocytes lipolysis, and the phosphorylation of HSL on Ser559/660 is crucial for its activation (Anthonsen et al., 1998). Here we observed that the phosphorylation of HSL was enhanced by C26 medium, which was successfully inhibited in the presence of PDTC (**Figures 5C,D**).

Likewise, the protective effect of PDTC was observed in TNFα-induced lipolysis of mature 3T3-L1 adipocytes. In detail, PDTC inhibited TNFα-induced decrease of lipid in mature 3T3-L1 adipocytes and increase of glycerol release in culture medium of mature 3T3-L1 adipocytes (**Figures 5E,F**). Compared to control group, the glycerol content increased to 170% in culture medium of mature 3T3-L1 adipocytes treated with TNFα, while decreased to 150, 137, and 120% in the presence of PDTC (10, 30, and 100 μM, respectively), which was in a concentration dependent manner. Here we observed that the phosphorylation of HSL was enhanced by TNFα, which was then successfully inhibited in the presence of PDTC (**Figures 5G,H**). Furthermore, the concentration of PDTC and TNFα used in these experiments had no effect on 3T3-L1 mature adipocytes viability (data not shown). These results demonstrated that PDTC inhibited the lipolysis process in 3T3-L1 mature adipocytes in cancer cachexia condition.

### Effect of PDTC on Inflammatory Signaling and Energy Metabolism of 3T3-L1 Mature Adipocyte *in Vitro*

**FIGURE 6:**
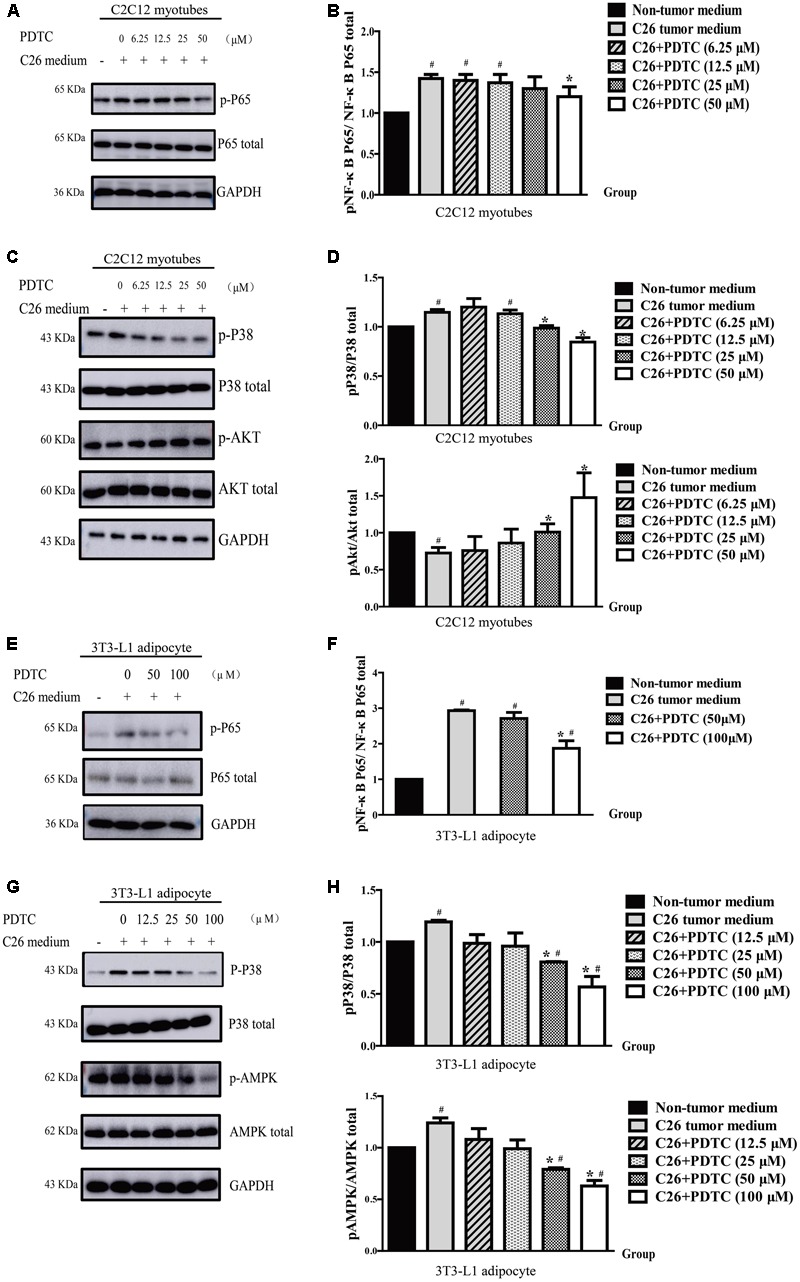
Pyrrolidine dithiocarbamate influenced different pathways in C2C12 myotubes and 3T3-L1 mature adipocyte. The C2C12 myotubes atrophy **(A–D)** and 3T3-L1 adipocyte lipolysis **(E–H)** in cancer cachexia model *in vitro* was induced by C26-tumor medium (1:1 dilution with fresh normal medium) for 48 h. **(A)** Representative Western blot of phosphorylated p65 and total p65 in C2C12 myotubes cachexia model induced with C26-tumor medium. **(B)** The quantification of A. **(C)** Representative Western blot of phosphorylated p38 MAPK, total p38 MAPK, phosphorylated AKT and total AKT in C2C12 myotubes cachexia model induced with C26-tumor medium. **(D)** The quantification of C. **(E)** Representative Western blot of phosphorylated p65 and total p65 in 3T3-L1 adipocyte cachexia model induced with C26-tumor medium. **(F)** The quantification of E. **(G)** Representative Western blot of phosphorylated p38 MAPK, total p38 MAPK, phosphorylated AMPK, and total AMPK in 3T3-L1 adipocyte cachexia model induced with C26-tumor medium. **(H)** The quantification of I. Data presented are the mean ± SE of three independent experiments. ^#^Versus non-tumor medium or control group; ^∗^versus C26-tumor medium or TNFα treatment group. ^#^*p* < 0.05, ^##^*p* < 0.01, ^###^*p* < 0.001, ^∗^*p* < 0.05, ^∗∗^*p* < 0.01.

Previous studies demonstrated that NF-κB influenced human fat cell lipolysis and the expression pro-inflammatory adipokines (Laurencikiene et al., 2007; Hatano et al., 2014), so we wondered whether PDTC would affect the NF-κB signaling in lipolysis of mature 3T3-L1 adipocytes *in vitro*. Western Blot results showed that PDTC was able to inhibit the enhanced phosphorylation of NF-κB induced by C26 medium slightly (**Figures 6E,F**). Interestingly, we found that PDTC significantly reduced the phosphorylation of p38 and AMPK which were enhanced by C26 medium (**Figures 6G,H**). Overall, these results demonstrated that PDTC was able to inhibit lipolysis by suppressing p38 MAPK signaling and AMPK signaling.

## Discussion

Cancer cachexia, characterized by severe wasting of muscle and fat, systemic inflammation, and energy metabolism hyperthyroidism, contributes to high mortality rate of cancer patients, especially for advanced solid tumor. There are a few clinical treatments to rescue cancer cachexia symptom, such as nutritional supplemental, which had been proven to be non-effective for cancer cachexia patients (Lainscak et al., 2008). Therefore, the discovery of effective anti-cancer cachexia drugs is very urgent and important. PDTC was reported to attenuate the development of cancer cachexia in mice bearing C26 and LLC tumor and in *APC^Min/+^* mouse (Nai et al., 2007; Puppa et al., 2014b; Narsale et al., 2016). However, the mechanism of PDTC on relieving cancer cachexia is largely unclear. Better understanding of the signaling pathways PDTC participating will allow the identification of potential therapeutic targets and is beneficial for the therapeutic of cancer cachexia.

Here we found that PDTC attenuated cancer cachexia symptom in C26 tumor bearing mice *in vivo* in our laboratory system, which was in consistence with previous studies (Nai et al., 2007; Puppa et al., 2014b; Narsale et al., 2016). PDTC significantly reduced body weight loss without influencing the tumor growth (**Figure 2**). Importantly, PDTC significantly attenuated the wasting of skeletal muscle and adipose tissue of the tumor-bearing mice as evidenced by the increased GA mass and myofiber area as well as the increased eWAT weight and diameter of adipocyte cells after PDTC treatment (**Figure 3**). Interestingly, these effects were further recaptured in *in vitro* system. PDTC blockaded C2C12 myotubes atrophy and 3T3-L1 mature adipose lipolysis induced by C26 tumor media or TNFα, suggesting that PDTC have direct effect on signaling pathways that mediated the wasting of skeletal muscle and adipose tissue (**Figures 4, 5**).

Skeletal muscle is the most obvious tissue affected by cancer cachexia and MHC is a preferred target of multiple pro-cachectic factors inducing muscle atrophy (Cosper and Leinwand, 2012; Umeki et al., 2015). In our study, we found that C26 medium or TNFα caused the decrease of MHC in mature C2C12 myotubes, and PDTC obviously reversed the downregulation of MHC. MyoD has been shown to drive the transcription of MHC (Meissner et al., 2007; Daou et al., 2013). Here we observed that PDTC treatment inhibited C26 medium-induced downregulation of MyoD, which might contribute to reverse the downregulation of MHC. In addition, the ubiquitin-dependent proteasome pathway has been reported to play important roles in muscle wasting process. The E3 ubiquitin ligase MuRF1 was involved in the degradation of MHC (Krawiec et al., 2005; White et al., 2011; Rom et al., 2015). Here we found that PDTC effectively reduced the expression of MuRF1, suggesting that PDTC also had effect on blocking the ubiquitin-dependent proteasome pathway. In our study, we observed that PDTC slightly inhibited the phosphorylation of p65 enhanced by C26 medium, suggesting other signaling pathways were employed by PDTC to relieve cancer cachexia. It is reported that the phosphorylation of AKT was inhibited in muscle atrophy (Quan-Jun et al., 2017), and the reduction of AKT phosphorylation led to increased MuRF1 transcription (Wadosky et al., 2014), so we wondered whether PDTC would affect the activation of AKT. Meaningfully, PDTC effectively increased the phosphorylation of AKT in C26 medium-treated C2C12 myotubes. Moreover, AKT signaling pathway also contributed to protein synthesis of skeletal muscle. Therefore, PDTC preserved the muscle mass by influencing the pathways of synthesis pathway and protein degradation. What’s more, the p38 MAPK signaling has been demonstrated to play important roles in skeletal muscle atrophy. Endotoxin-induced skeletal muscle wasting was reported to be through a p38 MAPK-dependent mechanism (Morales et al., 2015). Excessive fatty acid oxidation induces muscle atrophy in cancer cachexia by activating p38 MAPK pathway (Fukawa et al., 2016). Myostatin increased protein degradation and decreases protein synthesis of skeletal muscle by activation of the SMAD complex and by MAPKs and through PI3K/Akt pathway (Argiles et al., 2012). It has also been reported that p38 inhibitor could attenuate loss of skeletal muscle. P38 inhibitors, SB203580, blunted the expression of Atrogin1/MAFbx, and E3 ligases induced by TNF-α, and attenuated the protein degradation in C2C12 myotubes (Li et al., 2005). In addition, SB203580 also attenuated total protein degradation induced by TNF-α/IFN-γ and ANG II in murine myotubes (Eley et al., 2008). SB202190 (p38 inhibitors) administration blocks atrogin1/MAFbx upregulation and muscle protein loss in the muscle of LLC tumor-bearing mice (Zhang et al., 2011). In our study, we also found that C26 medium significantly enhanced the phosphorylation of p38 MAPK in C2C12 myotubes, and PDTC effectively inhibited this activation, indicating the effect of PDTC on relieving cancer cachexia was also through p38 MAPK signaling.

Previous researches on cancer cachexia mainly focus on muscle atrophy. But actually, fat is lost rapidly than skeletal muscle in cancer cachexia (Fouladiun et al., 2005; Das et al., 2011). In this study, we showed PDTC ameliorated cancer cachexia based on relieving not only muscle atrophy but also fat loss. The phosphorylation of HSL was upregulated by C26 medium to trigger adipocytes lipolysis, but PDTC effectively suppressed the increased phosphorylation of HSL, therefore inhibiting HSL-regulated adipocytes lipolysis. It is reported that HSL is a substrate for AMPK and activation of AMPK increases HSL phosphorylation (Garton and Yeaman, 1990; Holm et al., 2000; Holm, 2003; Carmen and Victor, 2006). It was also reported that the inhibitors of AMPK could alleviate lipolysis of fat. Ginsenoside Rh2 significantly activated AMPK and induced lipolysis in 3T3-L1 adipocytes, which was abolished by AMPK inhibitor treatment (Hwang et al., 2007). Compound C, an AMPK inhibitor, partially abrogated lipolysis of 3T3-L1 adipocytes by activating AMPK induced by thiacremonone (Kim et al., 2012). In addition, Compound C also attenuated lipolysis in isolated adipocytes induced by adrenaline (Koh et al., 2007). Here we found that PDTC significantly suppressed the phosphorylation of AMPK that was enhanced by C26 medium, suggesting the effect of PDTC on HSL might through AMPK. What’s more, the p38 MAPK signaling was also reported to phosphorylate HSL in pancreatic cancer exosome-induced adipose tissue lipolysis (Sagar et al., 2016). The inhibitors of p38 MAPK attributed to attenuate lipolysis of fat. Lipolysis induced by AM (adrenomedullin) and PC-exosomes could be attenuated in the presence of p38 MAPK inhibitor (SB203580) in 3T3-L1 and human adipocytes (Sagar et al., 2016). Interestingly, we also found that the phosphorylation of p38 MAPK was enhanced by C26 medium and PDTC effectively decreased this activation, suggesting p38 MAPK might be an important target for PDTC to exert its effect against cancer cachexia. In addition, the increased phosphorylation of p65 was modestly suppressed by PDTC in 3T3-L1 mature adipocytes lipolysis which was in consistence with the observation in muscle atrophy. Together, our study suggested that the protective effect of PDTC against C26 medium induced adipocytes lipolysis was not only targeting the NF-κB pathway, but also affecting the AMPK and p38 MAPK signaling pathways.

Up to now, the effects of reagents on attenuating cancer cachexia were usually evaluated by using *in vivo* animal models; however, these models are expensive and time consuming which definitely delay the development of drugs against cancer cachexia. Here we used C26 tumor medium or TNFα to induce atrophy of mature C2C12 myotubes and lipolysis of mature 3T3-L1 adipose cells *in vitro*, which mimics the wasting of skeletal muscle and adipose tissue *in vivo*. By using these models *in vitro*, we recapitulated the protective effect of PDTC on muscle atrophy and adipose lipolysis, suggesting that these two *in vitro* models could be used as a simple and reliable platform for the screening of anti-cancer cachexia drugs.

**FIGURE 7:**
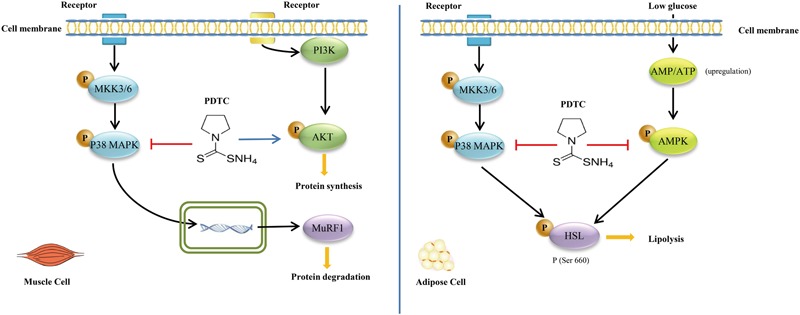
Pyrrolidine dithiocarbamate attenuates cancer cachexia by affecting both muscle and fat. PDTC regulated p38 MAPK signaling and AKT signaling to keep the mass of skeletal muscle, and played on p38 MAPK signaling and AMPK signaling to reduce the loss of fat.

In summary, our study showed that PDTC was sufficient to attenuate cancer cachexia-reduced loss of muscle and fat *in vitro* and *in vivo*. We further found that PDTC primarily influenced different pathways in different tissues. Specifically, PDTC regulated p38 MAPK signaling and AKT signaling to keep the mass of skeletal muscle, and regulated p38 MAPK signaling and AMPK signaling to reduce the loss of fat (**Figure 7**). Moreover, our study also established a simple and reliable *in vitro* cancer cachexia model for drug screening, which is definitely beneficial for identification of novel targets and development of new strategies for treatment of cancer cachexia.

## Author Contributions

Designed the experiments: XZ, XL, YF, CM, and YL. Performed the experiments: CM, YL, WZ, XC, and LF. Analysis and interpretation of data: CM and YL. Drafting the manuscript: XZ, XL, YF, and CM.

## Conflict of Interest Statement

The authors declare that the research was conducted in the absence of any commercial or financial relationships that could be construed as a potential conflict of interest.

## Abbreviations

C26: colon-26
HSL: hormone sensitive lipase
MHC: myosin heavy chain
PDTC: pyrrolidine dithiocarbamate
TG: triglycerides
